# Chemical Composition and Larvicidal Activities of the Himalayan Cedar, *Cedrus deodara* Essential Oil and Its Fractions Against the Diamondback Moth, *Plutella xylostella*

**DOI:** 10.1673/031.011.15701

**Published:** 2011-11-15

**Authors:** Abha Chaudhary, Prabha Sharma, Gireesh Nadda, Dhananjay Kumar Tewary, Bikram Singh

**Affiliations:** ^1^ Natural Plant Products Division, CSIR - Institute of Himalayan Bioresource Technology, Himachal Pradesh, India; ^2^ Entomology and Pesticide Residue Analysis Laboratory, Hill Area Tea Science Division, CSIR - Institute of Himalayan Bioresource Technology, Himachal Pradesh, India

**Keywords:** atlantones, biopesticide, essential oils, himachalenes, insecticidal activity

## Abstract

Plants and plant-derived materials play an extremely important role in pest management programs. Essential oil from wood chips of Himalayan Cedar, *Cedrus deodara* (Roxburgh) Don (Pinales: Pinaceae), was obtained by hydrodistillation and fractionated to pentane and acetonitrile from which himachalenes and atlantones enriched fractions were isolated. A total of forty compounds were identified from these fractions using GC and GC-MS analyses. Essential oils and fractions were evaluated for insecticidal activities against second instars of the diamondback moth, *Plutella xylostella* L. (Lepidoptera: Yponomeutidae), using a leaf dip method. All samples showed promising larvicidal activity against larvae of *P. xylostella.* The pentane fraction was the most toxic with a LC_50_ value of 287 µg/ml. The himachalenes enriched fraction was more toxic (LC_50_ = 362 µg/ml) than the atlantones enriched fraction (LC_50_ = 365 µg/ml). LC_50_ of crude oil was 425 µg/ml and acetonitrile fraction was LC_50_ = 815 µg/ml. The major constituents, himachalenes and atlantones, likely accounted for the insecticidal action. Present bioassay results revealed the potential for essential oil and different constituents of *C.* *deodara* as botanical larvicides for their use in pest management.

## Introduction

The diamondback moth, *Plutella xylostella* L. (Lepidoptera: Yponomeutidae), is an important and cosmopolitan pest of cruciferous crops (Harcourt 1962; Talekar and Shelton 1993; Gu et al. 2010). A major reason for the success of this pest is its remarkable ability to evolve insecticidal resistance (Cheng 1988; Sun et al. 2010). Several synthetic insecticides besides botanical and microbial control agents have been used for the control of this pest (Liu et al. 1982; Srinivasan and Krishnakumar 1982; Chaudhuri et al. 2001). The total annual cost for *P. xylostella* control throughout the world surpasses one billion US dollars (Talekar and Shelton 1993; Roux et al. 2007). The ill effects caused by chemical pesticides on health and environment, aside from resistance development in the pests, have fostered a need for the development of safer, lower-risk insecticidal agents. Natural plant products can be an excellent alternative source of novel insecticidal chemistries. With some exceptions, botanicals are considered to be less toxic to non-target species and more environmentally friendly because of their biodegradable nature (Copping 1996).

Plants are a virtually inexhaustible source of structurally diverse and biologically active substances; approximately 1800 plants have been reported to possess insecticidal properties (Jacobson 1982; Grainge et al. 1984). Plants are a good reservoir of eco-friendly allelochemicals. Extensive work has been done on bioactivity evaluation of extract/essential oil from various plants against important agricultural insect pests worldwide. Within the rich biodiversity of the Himalayas, there are abundant plant species, many of which are valued for their unique natural products and their biological and insecticidal properties (Tewary et al. 2005).

Himalayan Cedar, *Cedrus deodara* (Roxb. ex D. Don) (Pinales: Pinaceae), is found abundantly throughout the western Himalayas at altitudes of 1200–3000 m. Its essential oil has been reported to possess some activities against stored pests and houseflies (Singh et al. 1984; Singh and Rao 1985; Singh and Agrawal 1988; Singh et al. 1989). Its pesticidal activities are not reported against lepidopteron insect pests in the literature. Therefore, essential oil of *C. deodara* wood chip plant was selected for investigation against the diamondback moth. This study focuses on the chemical composition and larvicidal activities of *C. deodara* essential oil and its different chromatographic fractions against *P. xylostella.*

## Materials and Methods

### Insect cultures

*P. xylostella* used in this experimental study were collected from infested field crops and reared for more than 25 generations under laboratory conditions. Adult insects were allowed to lay eggs on one-week-old mustard plants grown in pots and caged inside wooden boxes. Larvae were removed from the mustard plants, transferred onto cabbage leaves, and kept in other boxes where development was completed. Adults were collected by aspirator and allowed to lay eggs on the mustard plants. Insects were reared and maintained at constant temperature of 25 ± 2° C, relative humidity of 60% ± 15, and photoperiod of 16:8 L:D. Second instar larvae were used in the experiments.

**Figure 1.**
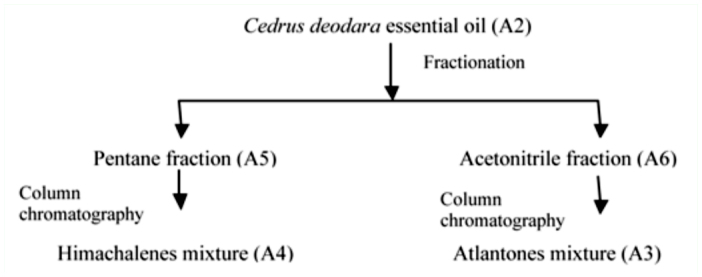
Fractionation protocol for essential oil of *Cedrus* *deodara.* High quality figures are available online.

### Plant material

Wood chips of *C. deodara* were collected in June 2007 from the forests of Mandi district in Himachal Pradesh, India, and voucher specimens (PLP 5969) were identified, processed, and deposited to the Herbarium of Institute of Himalayan Bioresource Technology (CSIR), in Palampur, India.

### Essential oil extraction and fractionation

The air—dried wood chips (1.5 kg) of *C. deodara* were subjected to hydrodistillation for six hours using a Clevenger apparatus. The oil was dried over anhydrous sodium sulphate. The oil (50 ml) was fractionated between pentane and acetonitrile (50 ml each × 3) (Figure 1). All the fractions were evaporated to dryness at 40° C under reduced pressure and stored in refrigerator at 4° C prior to analysis.

### Chromatography and characterization of essential oil fractions

Aliquots of pentane and acetonitrile fractions were subjected to chromatography over silica gel and eluted sequentially with nhexane/EtOAc gradients and finally with EtOAc. Fractions were tracked by thin layer chromatography and compounds with similar R_f_ values were pooled together to give sub-fractions. From pentane and acetonitrile fractions, mixtures of himachalenes and atlantones were isolated, respectively, which were identified by GC and GC-MS analyses. Various fractions of essential oil were designated as A_2_–A_6_ (A_2_: crude oil; A_3_: atlantone enriched fraction; A_4_: himachalene enriched fraction; A_5_: pentane fraction; A_6_: acetonitrile fraction).

### Analysis of volatile compounds of essential oil and its fractions

Analysis of the samples was performed on GC 2010 Shimazdu Gas Chromatograph (Shimadzu, www.shimadzu.com) equipped with an FID detector and a carbowax phase BP-20 capillary column (30 m × 0.25 mm i.d. with film thickness 0.25 µm). Nitrogen was used as a carrier gas with a flow rate 1.0 ml/minute. Oven temperature was programmed from 40–220° C at 4° C/min with a four min hold at 40° C and a 15 min hold at 220° C. Injector and interface temperatures were each 250° C for both. Ion source temperature was 200° C. The 20 µl sample was dissolved in 2 ml GC grade dichloromethane; sample injection volume was 2 µl.

GC-MS analysis was conducted on a Shimadzu QP2010 GC-MS system with 2010 GC. A carbowax phase BP-20 capillary column (30m × 0.25 mm i.d. with film thickness 0.25 µm) was used with helium as a carrier gas at a flow rate of 1.1 ml/min on split mode (1:50), using the same conditions as above. Relative percentages were calculated from the FID from the automated integrator. Kovats indices (KI) of the compounds relative to a mixture of n-alkanes (C_8_–C_23_) were calculated. Identification of compounds was first attempted using the mass spectral libraries Wiley 7 and NIST 02 (McLafferty 1989; Stein 1990). Corroboration of the identification was then conducted by matching the mass spectra of compounds with those present in the literature (Adams 1995; Jennings and Shibamoto 1980), and finally by matching the KI of the compounds reported on a column having an equivalent binding phase.

### Insecticidal activity

Essential oil and its various fractions (A_2_–A_6_) were screened for insecticidal activity at higher concentrations (10000, 5000, 2500, and 1250 µg/ml) using leaf dip method. Briefly, 200 mg of the samples were dissolved in three ml acetone and then diluted to a volume of 20 ml in distilled water containing 0.05% Triton X-100 LR spreader (SD Fine-Chem Limited, www.sdfine.com). It was serially diluted with distilled water containing 0.05% Triton X-100 to obtain lower concentrations. Based on the preliminary toxicity results, stock solutions (3000 µg/ml) of samples were prepared using the above method for dose response bioassay studies. Eight different concentrations (300023.4 µg/ml) of test samples were prepared by serial dilution in distilled water containing 0.05% Triton X-100. The prepared concentrations were poured in glass Petri dishes. Distilled water containing 0.05% Triton X-100 and 15% acetone was used as control.

Three cabbage leaf disks (6 cm diameter) were cut and dipped in either individual test or control solutions for 30 seconds.The solution from the disks was allowed to dry at room temperature. Ten second instar larvae of *P. xylostella*, starved for 3–4 hours, were transferred to treatment and control leaf disks kept on the moist filter paper in Petri dishes. Petri dishes were then sealed using parafilm. Treated and control Petri dishes were kept at 25 ± 2°C, 60 ± 15% relative humidity, and a photoperiod of 16:8 L:D for observations. Moisture build up inside the Petri dishes, if any had accumulated, was blotted using tissue paper and Petri dishes were resealed. Observations on mortalities were recorded 48 hours after the treatment was given. Larvae that did not show movements when probed with a camel hairbrush were considered dead. The experiment was repeated three times with three replications and pooled data were analyzed.

### Statistical analysis

Larval mortality was converted to percent mortality and corrected for control mortality using Abbott's formula (1925). Data were analyzed using EPA Probit Analysis Program version 1.5 for calculating LC_50/90_ values.

**Table 1.**
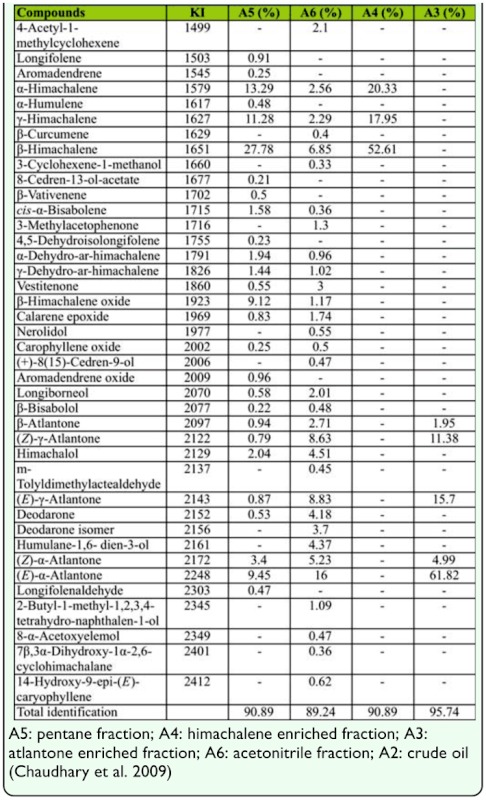
Chemical constituents of essential oil and its fractions obtained from *Cedrus deodara* wood chips.

## Results and Discussion

### Chemical constituents of essential oil

Essential oil was obtained by hydrodistillation with a 0.98% yield (w/w on dry weight basis). Pentane, acetonitrile, atlantone enriched and himachalene enriched chromatographic fractions were obtained. A total of forty compounds were identified from these fractions using GC and GC-MS analyses. The identified constituents, percentage composition, and their KI values are shown in Table 1. In an earlier study, Chaudhary et al. (2009) reported 36 constituents in oil of woodchips of *C. deodara* using GC and GC-MS analyses. In the pentane fraction, 27 compounds were identified representing 90.89% of the constituents detected. A total of 11 sesquiterpene hydrocarbons and 16 oxygenated sesquiterpenes were identified constituting 59.68 and 31.21%, respectively. In the acetonitrile fraction, 31 compounds were identified representing 89.24% of the constituents detected assigning to three different classes: oxygenated monoterpene (3.73%), sesquiterpene hydrocarbons (14.44%), and oxygenated sesquiterpenes (71.07%). The major constituents in the pentane fraction were himachalenes (52.35%) and atlantones (15.45%). The other constituents were himachalene oxide (9.12%), himachalol (2.04%), α-dehydro-*ar-*himachalene (1.94%), c*is*-α-bisabolene (1.58%), and γ-dehydro-*ar*-himachalene (1.44%). The major constituents in the acetonitrile fraction were atlantones (41.40%) followed by himachalenes (11.70%). Further chromatography led to an increase in the percentage of himachalenes and atlantones from pentane and acetonitrile fraction to 90.89 and 95.74%, respectively.

Generally, the *Cedrus* oils contain high percentages of the himachalenes. In another species, *C.* *atlantica*, essential oil is reported to contain 67% himachalenes as the major component (Boudarene et al. 2002). α-Pinene is commonly reported in *C. atlantica* needle oil, comprising more than 37% of the total essential oil. *C. libani* is rich in himachalenes (∼ 42%) in wood extract, α-pinene (∼ 24%), and caryophyllene (∼ 7%) in the needle oil (Fleisher 2000). Himachalenes (68.52%), atlantones (15.02%), and himachalol (1.00%) in the fractions of oil were similar to those previously reported with variation in percent composition (Nigam et al. 1990). There is a significantly higher percentage of atlantone (∼ 67%) from the extract of *C. deodara* as compared to other species, where they constitute less than 10% of the total oil or extract. Essential oil content could differ greatly even in the same genus, as well as in different plant parts (Duquesnoy et al. 2006; Salido et al. 2002; Cavaleiro et al. 2002). In our study, there was an absence of some major constituents like cedrene and cedrol, previously reported by Nigam et al. (1990). The chemical composition of an essential oil could also vary depending on geographical area, collecting season, distillation technique, stage of the plant part used for distillation, and presence of chemotypes and chemical races within the same species.

**Figure 2.**
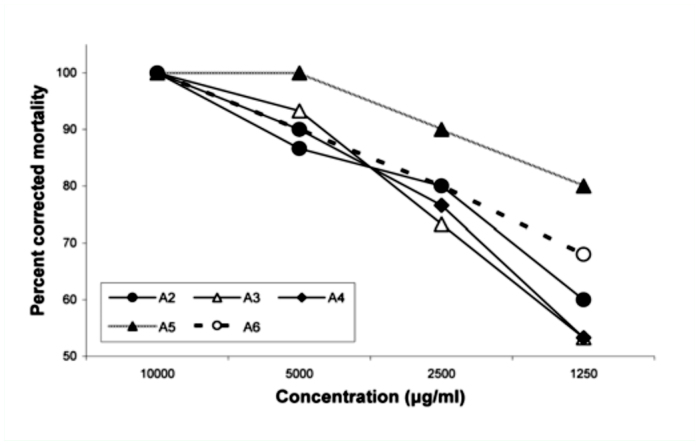
Preliminary screening of *Cedrus deodara* wood chip oil and its different fractions against larvae of *P.* *xylostella* (48 hours). High quality figures are available online.

### Insecticidal activity

Mortalities of second instar larvae of *P. xylostella* that were exposed to higher concentrations of essential oil and fractions (A_2_–A_6_) of *C. deodara* during the preliminary screening are presented in Figure 2. The samples efficiently killed the larval stages, and activity increased with increasing concentrations. The highest concentration (10,000 µg/ml) resulted in ∼ 100% mortality. The lethal concentration (LC_50_ and LC_90_) values along with other statistical parameters based on the dose response bioassay studies are presented in Table 2. The volatile constituents of pentane fraction A_5_ exhibited maximum larvicidal activity with minimum LC_50_ (287.06 µg/ml) and LC_90_ (2253.33 µg/ml) values, whereas acetonitrile fraction was the least toxic (LC_50_ = 815.48 µg/ml; LC_90_ = 5720.00 µg/ml).

**Figure 3.**
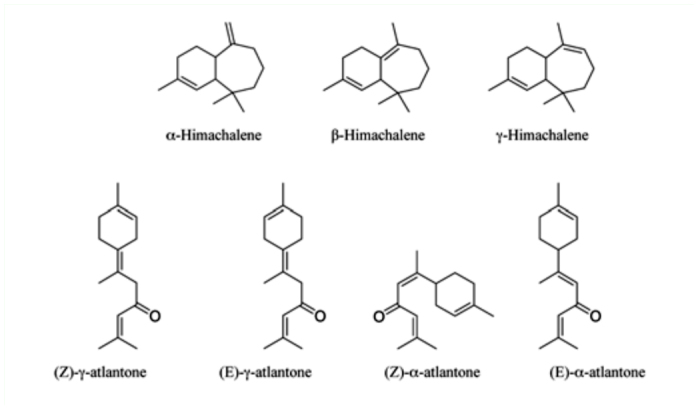
Structures of major constituents of *Cedrus deodara.* High quality figures are available online.

Larvicidal potential of the samples after 48 hours of exposure time was found in the following order: A_5_>A_4_>A_3_>A_2_>A_6_ on the basis of their LC_50_ and LC_9_o values. Himachalenes and atlantones enriched fractions exhibiting LC_50_ of 361.84 and 365.12 g/ml, respectively. The major constituents in the pentane fraction were himachalenes (52.35%) and atlantones (15.45%) (Figure 3). The other constituents were himachalene oxide (9.12%), himachalol (2.04%), α-dehydro-*ar*-himachalene (1.94%), *cis*-α-bisabolene (1.58%), and γ-dehydro-*ar-*himachalene (1.44%). It was observed that individual himachalenes and atlantones enriched fractions were less toxic than in the mixture.

**Table 2.**
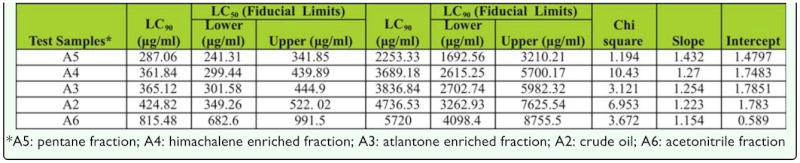
LC_50_ and LC_50_ values of essential oil and its fractions from *Cedrus deodara* against *Plutella xylostella*

In the present study, *C. deodara* essential oil and chromatographic fractions showed good larvicidal activities against *P. xylostella.* However, there is no available literature on the larvicidal potential of *C. deodara* oil and its fractions against this lepidopteron pest. Himalayan cedar wood oil and its constituents are reported to show insecticidal activities against some other insect pests like the Graham bean weevil (*Callosobruchus analis*), rice weevil (*Sitophilus oryzae*), housefly (*Musca domestica*), and the Chinese bean weevil (*Callosobruchus* chinensis) (Raguraman and Singh 1997; Singh et al. 1989; Singh and Agarwal 1988; Singh and Rao 1985). Further, *C. deodara* oil is reported to show toxicity against *Lymnaea acuminata* when combined with extracts of *Azadirachta indica* and *Embelia ribes* (Rao and Singh 2001). It is well known that the sensitivity of different insect species could be quite different for the same substance, and that insects vary widely in their responses to secondary plant products. Some plant-based compounds possess repellent and feeding and/or oviposition deterrent properties (Ibrahim et al. 2001). A number of research results have also been published on the use of plant based compounds for controlling herbivorous insects.

The insecticidal potential of essential oils for developing promising insect control agents has been emphasized in a number of recent reports (Adebayo et al. 1999; Gbolade et al. 2000). In studies of evaluating essential oils against lepidopteron larvae, patchouli oil was found to be the most toxic to oblique banded leafroller (*Choristoneura rosaceana*) larvae (LC_50_ = 2.8 µl/ml and LD_50_ = 8.0 µg/insect, whereas garlic oil was the most toxic to *Trichoplusia ni* larvae (LC_50_ = 3.3 µl/ml and LD_50_ = 22.7 µg/insect), followed by patchouli oil and lemongrass oil (Machial et al. 2010). In another study, essential oils of *Thymus vulgaris* (LC_50_ = 4.8 mg/ml), *Syzygium aromaticum* (LC_50_ = 6.0 mg/ml), *Cymbopogon citrates* (LC_50_ = 7.7 mg/ml), *Cinnamomum cassia* (LC_50_ = 8.5 mg/ml), *Cymbopogon nardus* (LC_50_ = 10.1 mg/ml) were found toxic to *T. ni* larvae in residual bioassays (Jiang et al. 2010). *Laurus azorica* and *Juniperus brevifolia* leaf essential oil caused 93.3% and 46.7% mortality of fourth-instar larvae of the armyworm, *Pseudaletia unipuncta*, and all essential oils significantly inhibited larval growth after five days of feeding on the treatment diet (Rosa et al. 2010).

Naturally occurring insecticide prototypes are in great demand at a global level to manage insect resistance and foster environmental health. In the present study, essential oil and fractions of *C. deodara* were found to possess larvicidal activities against *P. xylostella.* Therefore, these agents can be utilized for sustainable pest management. These fractions could be explored for their utilization in the botanical formulations either alone or in different combinations. However, further studies should be conducted to evaluate cost, efficacy, and safety of essential oil and enriched fractions on a wide range of pests. Results of this study could be helpful in further research for selection/identification or synthesis of semi-synthetic, newer, and more selective larvicidal compounds to develop lower-risk pesticides for use in integrated pest management packages. The use of essential oil in pest management could be of both economic and ecological benefit.
